# Machine Learning-Based Radiomics Nomogram for Detecting Extramural Venous Invasion in Rectal Cancer

**DOI:** 10.3389/fonc.2021.610338

**Published:** 2021-03-26

**Authors:** Siye Liu, Xiaoping Yu, Songhua Yang, Pingsheng Hu, Yingbin Hu, Xiaoyan Chen, Yilin Li, Zhe Zhang, Cheng Li, Qiang Lu

**Affiliations:** ^1^ Department of Diagnostic Radiology, Hunan Cancer Hospital and the Affiliated Cancer Hospital of Xiangya School of Medicine, Central South University, Changsha, China; ^2^ Department of Intestinal Oncology Surgery, Hunan Cancer Hospital and the Affiliated Cancer Hospital of Xiangya School of Medicine, Central South University, Changsha, China; ^3^ Department of Pathology, Hunan Cancer Hospital, Changsha, China

**Keywords:** rectal cancer, extramural venous invasion, radiomics, magnetic resonance imaging, computed tomography, prediction

## Abstract

**Objective:**

To establish and validate a radiomics nomogram based on the features of the primary tumor for predicting preoperative pathological extramural venous invasion (EMVI) in rectal cancer using machine learning.

**Methods:**

The clinical and imaging data of 281 patients with primary rectal cancer from April 2012 to May 2018 were retrospectively analyzed. All the patients were divided into a training set (n = 198) and a test set (n = 83) respectively. The radiomics features of the primary tumor were extracted from the enhanced computed tomography (CT), the T2-weighted imaging (T2WI) and the gadolinium contrast-enhanced T1-weighted imaging (CE-TIWI) of each patient. One optimal radiomics signature extracted from each modal image was generated by receiver operating characteristic (ROC) curve analysis after dimensionality reduction. Three kinds of models were constructed based on training set, including the clinical model (the optimal radiomics signature combining with the clinical features), the magnetic resonance imaging model (the optimal radiomics signature combining with the mrEMVI status) and the integrated model (the optimal radiomics signature combining with both the clinical features and the mrEMVI status). Finally, the optimal model was selected to create a radiomics nomogram. The performance of the nomogram to evaluate clinical efficacy was verified by ROC curves and decision curve analysis curves.

**Results:**

The radiomics signature constructed based on T2WI showed the best performance, with an AUC value of 0.717, a sensitivity of 0.742 and a specificity of 0.621. The radiomics nomogram had the highest prediction efficiency, of which the AUC was 0.863, the sensitivity was 0.774 and the specificity was 0.801.

**Conclusion:**

The radiomics nomogram had the highest efficiency in predicting EMVI. This may help patients choose the best treatment strategy and may strengthen personalized treatment methods to further optimize the treatment effect.

## Introduction

Rectal cancer is one of the major causes of cancer-related mortality in the world, with a local recurrence rate of up to 30% related to the surgical technique (1). Local recurrence and metastasis are the main causes of death in patients with rectal cancer, and there is much evidence that extramural venous invasion (EMVI) is an independent predictor of local tumor recurrence, ectopic nodules, distant metastasis and overall mortality (2–4). Therefore, the early identification of EMVI is of great significance for the selection of treatment strategies.

At present, pathological examination is still the gold standard for evaluating the EMVI status of rectal cancer, but the pathological EMVI status can only be obtained after surgery, which is not conducive to early treatment decisions before surgery. Besides, preoperative neoadjuvant therapy may lead to the underestimation of EMVI status in postoperative pathological examination (5). In recent years, studies have shown that preoperative imaging can improve the prognosis of rectal cancer (6). Due to the advantages of high spatial resolution, magnetic resonance imaging (MRI) is an excellent imaging method to detect the adverse prognostic factors of rectal cancer, and it is a promising and repeatable technique for the identification of EMVI. Several studies have shown that MRI has medium to high sensitivity and specificity in detecting EMVI compared with pathological evaluation (7–9). However, it should be noted that MRI may not be able to correctly identify the invasion of small extramural and intramural vessels, which leads to the low sensitivity of conventional MRI in the evaluation of EMVI (10, 11). On the other hand, computed tomography (CT) can assess the entire abdomen, pelvis and chest, allowing for local staging and distant metastasis evaluation (12, 13). Accordingly, modern CT techniques are better suited than MRI to search for the local tumor extent and distant metastases in the same imaging session (14). It is well known that EMVI is associated with disease recurrence, especially in patients with distant metastasis to the liver (15). Therefore, to some extent, preoperative CT may help identify EMVI indirectly. However, the sensitivity of CT for identifying EMVI was low due to its low resolution in soft tissue (16).

Radiomics is a noninvasive and relatively cost-effective image evaluation technology (17, 18). At present, radiomics technology has been widely used in the field of rectal cancer for tumor staging, prognosis evaluation and metastasis prediction (19). However, to our knowledge, only Yu et al. have focused on predicting EMVI based on radiomics (20). But their results showed that their radiomics model had poor stability and low sensitivity, which may be resulted from their small amount of data and defects in the modeling method. In fact, predictive and prognostic models are an important part of radiology (21), and highly accurate and reliable models are needed to improve decision support in clinical practice. Machine learning can be helpful in this respect (22). Therefore, we hypothesized that a radiomics model constructed based on machine learning can improve the prediction accuracy of EMVI, thus enhancing the application of noninvasive and cost-effective radiomics in the preoperative prediction of EMVI.

The main purpose of this study was to construct and validate a radiomics nomogram using machine learning to provide a convenient and quick tool to accurately predict preoperative EMVI in clinical practice.

## Materials and Methods

### Patient Data

This retrospective study was approved by the ethics committee of our institution, and informed consent was not required. A total of 281 patients who underwent radical resection of rectal cancer from April 2012 to May 2018 were included in this study, and their preoperative clinical and imaging data were retrospectively analyzed. The inclusion criteria were as follows: (1) pathologically confirmed non-mucinous rectal adenocarcinoma after surgery, and (2) completed baseline MRI and CT examinations before surgery. The exclusion criteria were as follows: (1) received preoperative antitumor treatment for rectal cancer, (2) incomplete clinical, pathological or imaging data, or (3) poor quality of CT or MRI images. All patients were grouped into a training set (n = 198; between April 2012 and May 2016) and a test set (n = 83; between June 2016 and April 2018) at a ratio of 7:3. The training set was used to build the radiomics nomogram, and the test set was used for model validation.

### Image Acquisition

All patients were examined by abdominal CT and pelvic (rectum) MRI within 1 week before surgical operation. All MRI examinations were performed with a 3.0-T MRI scanner (Discovery 750W^®^, GE Healthcare, Waukesha, WI). The MRI sequences included high-resolution T2-weighted imaging (T2WI) (transverse, coronal and sagittal), T1-weighted imaging (T1WI) (transverse), diffusion-weighted imaging (DWI) (transverse) and gadolinium contrast-enhanced T1WI (CE-T1WI) (transverse) sequences. For venous phase CE-T1WI, the contrast agent gadodiamide (Omniscan^®^, GE Medical System, NJ) was intravenously administered at a dose of 0.1 mmol/kg of body weight with a flow rate of 3.5 ml/s using a power injector, followed by a bolus injection of 20 ml of normal saline. Plain and enhanced CT images were obtained using a 256-detector row CT scanner (Revolution Xtream^®^, GE Healthcare, Waukesha, WI). For enhanced CT imaging, the injection rate of the contrast medium (Omnipaque^®^ 350, GE Medical System, NJ) was 2.5 ml/s, and the scan was performed after a 50-s delay. The specific scanning parameters are provided in the additional materials.

### EMVI Assessment

Pathological reports were retrieved and reviewed to obtain EMVI status. Two experienced pathologists (pathologist A and pathologist B, both of whom had experience of more than 10 years in the diagnosis of rectal cancer and were blinded to the CT and MRI data) independently extracted the EMVI status from the report. The pathological definition of EMVI is the presence of tumor tissue in the endothelium-lined lumen, which is surrounded by smooth muscle edges or contains red blood cells (23). If there was conflicting information between the EMVI assessment and other descriptions in the pathological or surgical report, the two pathologists would discuss the case and reach a consensus. Finally, 125 EMVI-positive cases and 156 EMVI-negative cases were identified.

To distinguish pathological EMVI, we defined the EMVI status evaluated by MRI as mrEMVI. A five-point scoring system was used for the assessment of mrEMVI (2, 8). The details of scoring from 0 to 4 are described in the supporting materials. A score of 0-2 was defined as mrEMVI-negative, and a score of 3-4 was defined as mrEMVI-positive.

### Segmentation and Feature Extraction of the Primary Tumor

Region of interest (ROI) segmentation was performed on CT and MRI images by ITK-SNAP software (http://www.itksnap.org). For CT, segmentation was based on venous phase–enhanced images. While for MRI, segmentation was based on T2WI and CE-T1WI. The above segmentation was manually completed by two experienced radiologists (radiologist A and radiologist B, with experience of 5 and 12 years in tumor imaging, respectively) who were blinded to the pathological information. Then, the segmented image was imported into quantitative Analysis Kit (AK, version 1.2, GE Healthcare) for image preprocessing, including resampling the image to a 1 × 1 × 1 mm^3^ voxel size and standardizing the image gray level to a scale of 1 to 32 to eliminate the influence of anisotropy on the extracted features (24). The image gray-scale intensity level was discretized and normalized by down-sampling each image to 32 bins to reduce image noise. Using such fixed values and number of bins the image gray range was divided into equally spaced intervals. Therefore, the bin size and intensity resolution of the discretized volumes depended on the gray-scale value (i.e., four bin sizes for each gray-level). Then, 378 radiomics features were extracted from the preprocessed images of each mode, including histogram features, Form Factor features, gray level co-occurrence matrix (GLCM) features, run-length matrix (RLM) features and gray level size zone matrix (GLSZM) features. These features have been shown to be characteristic of cancer heterogeneity and may reflect changes in image structure (18). Details of the features are provided in the additional materials. In addition, to ensure the robustness of the extracted features, we used the most effective feature among different radiologists for manual segmentation. The Spearman rank correlation test was used to calculate the correlation coefficient (CC) of each feature between feature set A (from radiologist A) and feature set B (from radiologist B). Features with CC > 0.8 were considered robust features (25). The feature values in this study were the average values of feature set A and feature set B.

### Establishment and Evaluation of the Radiomics Signature

The existence of a “curse of dimensionality” usually makes data simplification or feature selection necessary to obtain meaningful results from pattern recognition analysis (26). Therefore, it is necessary to reduce the dimensionality of the extracted robust features. The process of dimensionality reduction consists of two steps. First, the minimum redundancy maximum correlation (mRMR) algorithm was used to reduce the dimensionality of the robust features of the training set. The purpose of the maximum correlation program is to select features that are most relevant to the EMVI state. At the same time, the minimum redundancy process ensures minimum redundancy between the selected features to obtain the optimal features with a high correlation and a low redundancy (27). After that, the least absolute shrinkage and selection operator (LASSO) algorithm was used to select the features for constructing the radiomics signature from the best feature sets. Finally, logistic regression was used to construct the radiomics signature. In addition, to quantify the accuracy of the signature constructed by different modes (enhanced CT, T2WI and CE-T1WI), we calculated the EMVI-positive probability score of each case using the radiomics formula of the training set, which was defined as the rad score. Moreover, a receiver operating characteristic (ROC) curve was used to visualize the experimental results using data from the test set, and the area under the curve (AUC) values of the training and test sets were calculated to quantify the prediction performance of the radiomics signature. In addition, to further select the optimal radiomics signature, we used the DeLong test to compare the performance of signatures from different modes.

### Construction and Evaluation of the Radiomics Nomogram

The clinical characteristics of the training set, including gender, age, tumor location, carcinoembryonic antigen (CEA), degree of pathological differentiation, and mrEMVI, were analyzed by stepwise logistic regression to select the independent clinical predictors of EMVI. According to the best radiomics signature selected by the DeLong test, different joint models were built using support vector machine (SVM) combined with each independent predictor. SVM is a kind of supervised learning model that is commonly used in pattern recognition, classification and regression analysis. In recent years, it has been successfully applied to the diagnosis, prognosis prediction and treatment of various diseases (28).

To compare the performance of each model, we use the ROC curve to evaluate the performance of the model in the training set and used the data of the test set for verification. Finally, the optimal model was selected to generate a visual radiomics nomogram. The Hosmer-Lemeshow test was used to analyze the goodness of fit of the nomogram. The calibration curve was used to measure the consistency between the predicted EMVI probability and the EMVI probability. In addition, to evaluate the clinical efficacy of the nomogram, we used the nomogram to calculate the prediction score of EMVI for each patient. Taking the best cutoff value corresponding to the Youden index threshold of the ROC curve of training set as the classification point (29), all patients were divided into a low-risk group and a high-risk group according to the prediction score, and the number of patients with EMVI-positive between the two groups was compared.

### Statistical Analysis

All statistical analyses in the present study were performed with SPSS (version 25.0), R 3.5.1 and Python 3.5.6. The Kolmogorov–Smirnov test was used for the normality testing of the measurement data. The normally distributed data were evaluated using the independent sample t-test, whereas the nonnormally distributed data were evaluated using the Mann–Whitney U test. The differences between categorical variables were tested by the chi-square test. ICC estimates and their 95% confident intervals were calculated using SPSS statistical package version 25 based on a mean-rating (k = 2), absolute-agreement, 2-way mixed-effects model. A two-tailed p-value < 0.05 was regarded as significantly different.

## Results

### Patient Clinical Data

**Figure 1:**
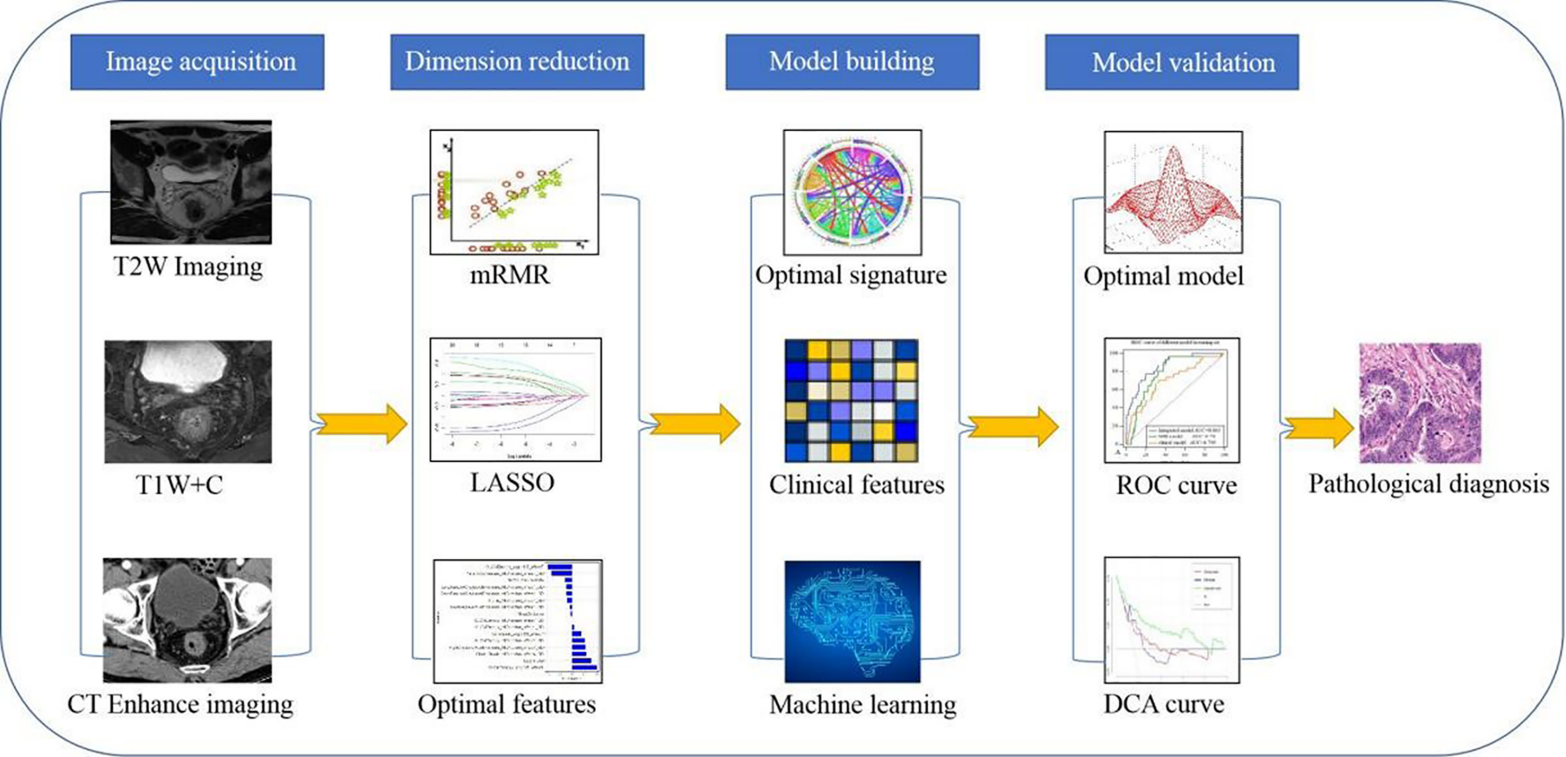
Research flow chart of the radiomics model.

The research flow chart is shown in **Figure 1**. There were no significant differences in any of clinical features between the training and test sets, as shown in **Table 1**. There were significant differences in lymph node metastasis and mrEMVI between patients who were EMVI-negative and patients who were EMVI-positive in the training and test sets. See **Table 2** for details.

**Table 1:** Descriptive statistics of the two sets.

Variables	Level	Training set (n = 198)	Test set (n = 83)	*P* value
Gender (n, %)	Male	79 (39.9)	41 (49.4)	0.181
	Female	119 (60.1)	42 (50.6)	
Age (year)	Mean (sd)	59.3 (10.2)	57.6 (10.8)	0.227
mrEMVI (n, %)	No	125 (63.1)	51 (61.5)	0.929
	Yes	73 (36.9)	32 (38.5)	
Tumor location (n, %)	Low-rectum	75 (37.9)	27 (32.5)	0.288
	Mid-rectum	85 (42.9)	44 (53)	
	High-rectum	38 (19.2)	12 (14.5)	
MRI LN status (n, %)	N0	99 (50)	42 (50.6)	1.000
	N1-2	99 (50)	41 (49.4)	
CEA (n, %)	Normal	143 (72.2)	62 (74.7)	0.78
	Abnormal	55 (27.8)	21 (25.3)	
Degree of pathological differentiation (n, %)	Low	24 (12.1)	9 (10.8)	0.64
	Medium	156 (78.8)	69 (83.1)	
	High	18 (9.1)	5 (6.0)	

mrEMVI, magnetic resonance extramural venous invasion.

**Table 2:** Clinical characteristics of the training and test sets.

Variables	Level	Training set (n = 198)			Test set (n = 83)		
		EMVI-negative (n = 110)	EMVI-positive(n = 88)	*P* value	EMVI-negative(n = 46)	EMVI-positive(n = 37)	*P* value
Gender (n, %)	Male	46 (41.8)	33 (37.5)	0.638	24 (52.2)	17 (45.9)	0.731
	Female	64 (58.2)	55 (62.5)		22 (47.8)	20 (54.1)	
Age (year)	Mean (sd)	59.2 (10.4)	59.3 (10.1)	0.974	57.2 (10.7)	58.1 (11)	0.709
mrEMVI (n, %)	Negative	96 (86.7)	29 (32.9)	<0.001*	35 (76.1)	16 (43.2)	0.002*
	Positive	14 (13.3)	59 (67.1)		11 (23.9)	21 (56.8)	
Location (n, %)	Low-rectum	46 (40.9)	27 (30.7)	0.194	19 (41.3)	8 (21.6)	0.162
	Mid-rectum	51 (46.4)	43 (48.9)		21 (45.7)	23 (62.2)	
	High-rectum	14 (12.7)	18 (20.5)		6 (13)	6 (16.2)	
MRI LN status (n, %)	N0	70 (63.6)	29 (33)	<0.001*	32 (69.6)	10 (27)	<0.001*
	N1-2	40 (36.4)	59 (67)		14 (30.4)	27 (73)	
CEA (n, %)	Normal	85 (77.3)	58 (65.9)	0.106	34 (73.9)	22 (59.5)	0.245
	Abnormal	25 (22.7)	30 (34.1)		12 (26.1)	15 (40.5)	
Degree of pathological differentiation (n, %)	Low	8 (7.3)	12 (13.6)	0.197	3 (6.5)	6 (16.2)	0.368
	Medium	89 (80.9)	70 (79.5)		40 (87)	29 (78.4)	
	High	13 (11.8)	6 (6.8)		3 (6.5)	2 (5.4)	

*P < 0.05. mrEMVI, magnetic resonance extramural venous invasion. EMVI, pathological extramural venous invasion.

### Diagnostic Performance of the Radiomics Signature

**Figure 2:**
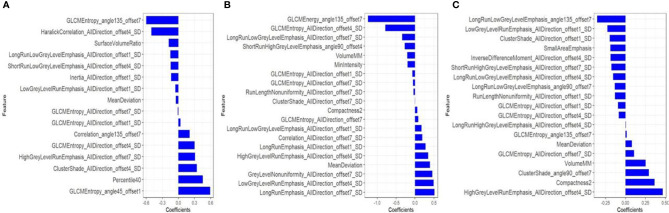
After dimensionality reduction by mRMR and LASSO, 16, 20 and 19 radiomics features were finally selected from CT-enhanced images **(A)**, T2WI **(B)** and CE-T1WI **(C)** to construct a radiomics signature. The blue bar indicates the weight value of the radiomics features.

**Figure 3:**
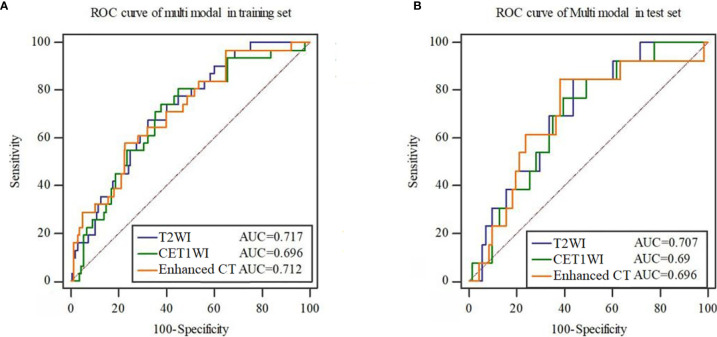
ROC curves of the radiomics signature constructed by each mode in the training set **(A)** and test set **(B)**.

**Figure 4:**
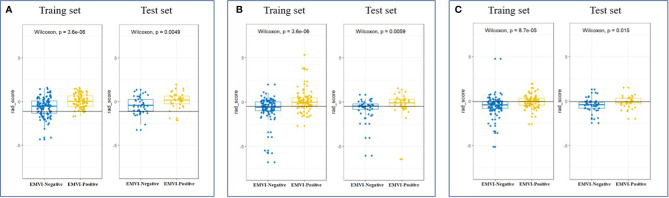
Scatter plot between EMVI-negative (blue dots) and EMVI-positive (yellow dots) rad scores calculated by radiomics signatures constructed by T2WI **(A)**, CE-T1WI **(B)** and CT-enhanced images **(C)** in the training and test sets.

As is shown in **Figure 2**, the remaining 16, 20 and 19 features after dimensionality reduction were extracted from enhanced CT, T2WI and CE-T1WI images respectively. The performance of the radiomics signature based on these features both in the training set and in the test set are as shown in **Figure 3**. The DeLong test showed that there was no significant difference in the AUC values among the three radiomics signatures in the training set and the test set (P > 0.05). Therefore, we selected the signature constructed by T2WI as the optimal signature for the construction of the joint model. Acquiring T2WI image can avoid both radiation damage and medical risk caused by contrast medium. In addition, the rad score was calculated based on each signature model in the training set and test set, and there were significant differences in the rad score between the EMVI-positive and EMVI-negative groups (*P* < 0.05), as shown in **Figure 4**. Details of the construction of the radiomics signature can be found in the supporting materials.

### Construction and Performance Evaluation of the Radiomics Nomogram

**Figure 5:**
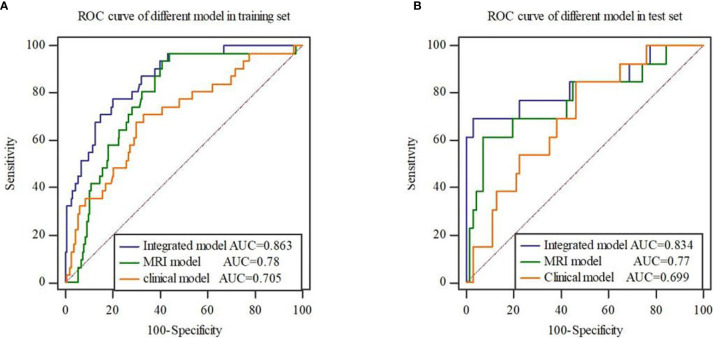
ROC curves of three models in the training set **(A)** and test set **(B)**. The results show that the integrated model has the highest AUC value.

**Figure 6:**
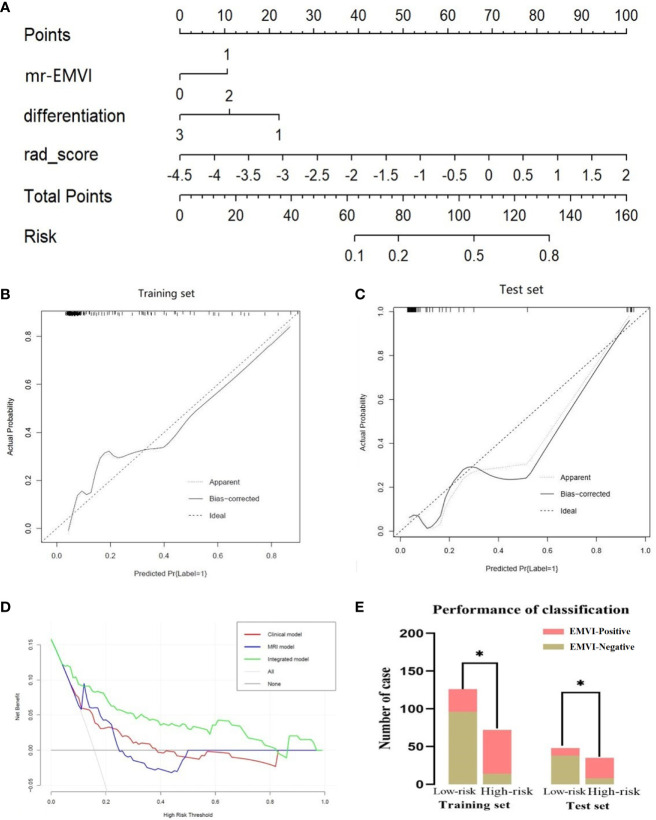
Radiomics nomogram for detecting EMVI **(A)**. In the nomogram, a vertical line was drawn according to the value of the rad score to determine the corresponding value of points. The points of mrEMVI and differentiation stage can also be determined in the same way. The total points were the sum of the three points above. Finally, a vertical line was drawn according to the value of the total points to determine the probability of EMVI. The calibration curve of the radiomics nomogram for EMVI in the training set **(B)** and test set **(C)**. A dashed line indicated the reference line where an ideal nomogram would lie. A dotted line indicated the performance of the nomogram, while the solid line indicated bias correction in the nomogram. DCA curve **(D)** for the integrated model, MRI model and clinical model predicting EMVI in the dataset. The graphs showed that the integrated model had the greatest net benefit. The risk classification performance of the integrated model in the training and test set **(E)**. *P < 0.05.

Stepwise logistic regression analysis showed that mrEMVI, degree of pathological differentiation and radiomics signature were independent predictors of EMVI, as shown in **Table 3**. Three models were constructed, including the clinical model (the optimal radiomics signature combining with the degree of pathological differentiation), the MRI model (the optimal radiomics signature combining with the mrEMVI status) and the integrated model (the optimal signature radiomics combining with both the degree of pathological differentiation and the mrEMVI status). The ROC curve showed that the AUC value of the integrated model was higher than those of other models and independent predictors in the training and test sets. The DeLong test showed that there was significant difference in the AUC values of the integrated model between MRI model and Clinical model in training set (P = 0.0129 and 0.0007), and in the test set, there were also significant differences (P = 0.0462 and 0.0159). Therefore, the radiomics nomogram was based on the integrated model, as shown in **Figures 5** and **6A** and **Table 4**. Finally, the Hosmer-Lemeshow test showed that the performance of the nomogram was not significantly different between the training and testing sets (P > 0.05). The calibration curve showed that the nomogram had better prediction performance (**Figures 6B, C**) and decision curve analysis (DCA) showed that the nomogram had the best clinical net benefit compared with the other models in the overall dataset (**Figure 6D**). Based on the classification value of the nomogram (cutoff = 0.463), the number of EMVI-positive cases in the low-risk group and the high-risk group was significantly different (**Figure 6E**).

**Table 3:** Stepwise logistic regression analysis of EMVI prediction.

Variable	Univariate logistic regression		Multivariate logistic regression	
	OR (95%CI)	*P* value	OR (95%CI)	*P* value
Gender (Male vs Female)	0.334 (0.123-1.721)	0.562	NA	NA
Age (per 1 increase)	0.467 (0.282-1.515)	0.628	NA	NA
mrEMVI (Negative vs Positive)	7.317 (3.086-17.35)	<0.001*	7.351(3.132-17.256	<0.001*
Location (Low vs Mid)	2.443 (1.227-5.872)	0.434	NA	NA
Location (Low vs High)	3.646 (1.643-6.563)	0.642	NA	NA
MRI LN status (N0 vs N1-2)	3.284 (2.341-6.732)	0.672	16.251 (6.549-40.326)	<0.0001*
CEA (per 1 increase)	1.557 (0.382-6.337)	0.537	NA	NA
Degree of pathological differentiation (Low vs Medium)	2.614 (1.475-14.37)	0.025*	2.665 (1.533-15.473)	0.014*
Degree of pathological differentiation (Low vs High)	0.935 (0.203-4.303)	0.031*	1.043 (0.382-5.482)	0.009*
Radiomics signature	1.463 (1.048-2.042	0.002*	1.535 (1.105-2.131)	0.01*

NA, not available as the variable, was not included in the multivariate logistic regression. mrEMVI, magnetic resonance extramural venous invasion; LN, lymph node. *P < 0.05.

**Table 4:** Diagnostic efficacy of different models and independent clinical predictors.

Group	Performance features	Integrated model	MRI model	Clinical model	mrEMVI	Degree of pathological differentiation
Training set	AUC	0.863	0.78	0.705	0.74	0.647
	Sensitivity	0.774	0.867	0.71	0.613	0.593
	Specificity	0.801	0.56	0.669	0.868	0.712
Test set	AUC	0.834	0.771	0.699	0.73	0.625
	Sensitivity	0.708	0.685	0.746	0.615	0.573
	Specificity	0.892	0.829	0.735	0.845	0.763

AUC, area under the curve. mrEMVI, magnetic resonance extramural venous invasion.

## Discussion

In this study, our results showed that there was no significant difference in the diagnostic performance of EMVI by three radiomics signatures based on CT-enhanced images, T2WI and CE-T1WI. T2WI is not involved in radiation damage and contrast-induced medical risk. Therefore, T2WI-based radiomics signature was selected in the present study to combine with clinical and mrEMVI data to build the radiomics nomogram. Our results also showed that the nomogram has the best predictive performance of the models. In view of the noninvasive and low-cost characteristics of radiomics technology, this may provide a new quantifiable tool for the preoperative evaluation of EMVI status.

In the diagnosis and treatment of rectal cancer, CT is mainly used for screening tumor metastasis, especially in the diagnosis of liver and lung metastases (30). However, due to the low signal-to-noise ratio of CT and the lack of ideal soft tissue resolution, there are great limitations in the visual evaluation of EMVI by CT (31). In this study, radiomics technology based on CT images can be used for EMVI evaluation, implying the advantages of radiomics technology (32). T2WI shows great performance in EMVI evaluation in rectal cancer (10) for its high resolution of soft tissue. Similarly, the radiomics signature based on T2WI showed the highest AUC value in this study. Although some prior studies have shown that CE-T1WI can display vascular structure and improve the diagnostic performance of EMVI for rectal cancer (33), there were no statistically significant differences observed between CE-T1WI and T2WI using radiomics signature for evaluation of EMVI in this study, which indicated that high-resolution T2WI may be more suitable for radiomics analysis than the CE-T1WI in predicting EMVI considering the cost, convenience and safety. In general, T2WI is more suitable for the radiomics analysis of rectal cancer, which may improve the clinical evaluation of EMVI.

The radiomics nomogram obtained in this study also shows superior performance in predicting EMVI. Our results are better than those of Brown et al, reporting that the sensitivity and specificity of conventional MRI for EMVI detection were 62% and 88% respectively (34), which may be benefit from the diagnostic performance of the radiomics signature, mrEMVI and clinical features. Previous studies have confirmed that the ability of mrEMVI was at least as good as that of routine histopathology (5). Resembly, our study showed that mrEMVI had higher diagnostic efficiency of EMVI than that of the radiomics signature, though the sensitivity of mrEMVI was significantly lower. Sohn et al. reported that the sensitivity of MRI in the evaluation of EMVI was only 28.2% (8), as the smallest vessel diameter that 3.0-T MRI can distinguish is 3 mm. Theoretically speaking, even if high-resolution T2WI is used, it is difficult to identify vessels with a diameter less than 3 mm (10, 35). In fact, it is very difficult to identify vessels on MRI, and it is usually necessary to compare different sequences at the same level in addition to intravenously using gadolinium contrast agent to confirm whether it is a vessel. Even if it can be confirmed as a blood vessel, many cases do not show the typical imaging features of vascular lumen expansion, irregular shape or the “flow empty” signal in the blood vessel replaced by the tumor signal. Therefore, even experienced radiologist can easily miss these atypical cases. However, the radiomics nomogram can be used to quantitatively evaluate EMVI through the radiomics analysis of primary tumors. In this process, only the delineation of the tumor area is through visual evaluation, which is obviously more accurate and easier than the all visual evaluation. Therefore, the radiomics technology has greater clinical advantages compared with the traditional visual assessment.

Compared to the same type of research by Yu et al. (20), the diagnostic efficiency of the nomogram in the training set was lower than that of the radiomics nomogram constructed in their study (AUC = 0.904), while the diagnostic efficiency of the nomogram in the test set was higher than that of theirs (AUC = 0.812), which indicated better stability of our nomogram. This may be caused by the different radiomics signature. In their study, the nomogram was constructed by the radiomics signature based on dynamic contrast-enhanced MRI. While in our research, the radiomics signature was on the basis of T2WI images. In fact, the use of contrast medium may affect the choice of radiomics features. The different doses of contrast medium and the permeability of tissue microvessels, which were related to the image enhancement effect (36), would change the distribution of pixels and then affect the stability of the whole model. In addition, Maxiao et al. (37) found that the radiomics model constructed by SVM had the best performance of the different machine learning methods in evaluating preoperative pathological features. Thus, the model generated by SVM in our study was more stable than that of Yu et al, which was built by logistic regression. In addition, the previous studies have shown that the accuracy of CT based super physiological vein diameter for predicting EMVI is 0.83 (38), which is equivalent to the accuracy of nomogram for identifying EMVI. However, compared with CT radiation damage, nomogram may be more suitable for clinical practice. Aysegul et al. Used changes in dimensions of superior recurrent vein (SRV) and inferior median vein (IMV) and ADC values were used to predict EMVI (39), and the AUC values were 0.851.0.893 and 0.664, respectively. Although the diagnostic efficiency of IMV was higher than that of the nomogram, but these indexes were based on CT examination, while the ADC value based on MRI examination was significantly lower than that of our nomogram. In fact, functional imaging such as DWI could not improve the efficiency of MRI in EMVI detection (35).

There were some limitations in this research. First, this study was retrospectively. However, eligible patients were consecutively retrieved from a prospective database that included all patients with rectal cancer in our hospital. Second, our data are limited to a single center study, so our results may not be extended to other medical centers. In the future, multicenter studies are needed to further verify the results of this study. Finally, this study did not analyze the correlation between the radiomics features and clinical features and lacked the interpretability of the radiomics features. In the future, we will further clarify the interpretability of the radiomics features.

In conclusion, T2WI-based radiomics technology was superior to CT and CE-TIWI in predicting the EMVI status in rectal cancer. At the same time, the radiomics nomogram combined with clinical features and mrEMVI was a convenient and noninvasive tool to predict the EMVI status accurately.

## Data Availability Statement

The raw data supporting the conclusions of this article will be made available by the authors, without undue reservation.

## Ethics Statement

The studies involving human participants were reviewed and approved by the medical ethics committee of Hunan Cancer Hospital. Written informed consent for participation was not required for this study in accordance with the national legislation and the institutional requirements.

## Author Contributions

All authors listed have made a substantial, direct, and intellectual contribution to the work and approved it for publication.

## Funding

This study was supported by the Provincial Key Clinical Specialty (Medical Imaging) Development Program from Health and Family Planning Commission of Hunan Province, China (contract grant number: 2015/43) , by Cancer Research Program of National Cancer Center (Grant Number: NCC2017A19), and by Nature Science Fund of Hunan Province, China (Project Number: 2020JJ4052).

## Conflict of Interest

The authors declare that the research was conducted in the absence of any commercial or financial relationships that could be construed as a potential conflict of interest.
