# Supplementary material for: Machine Learning-Based Radiomics Nomogram for Detecting Extramural Venous Invasion in Rectal Cancer
Source: Front Oncol. 2021 Mar 26;11:610338. doi: 10.3389/fonc.2021.610338 (PMC8033032; doi:10.3389/fonc.2021.610338)
Supplement: Supplementary file 1 [file DataSheet_1.docx]

**Machine learning-based radiomics nomogram for detecting extramural venous invasion in rectal cancer**

**I. Supplementary Methods**

**1. MRI classification of EMVI**

EMVI-score, an MRI-based preoperative grading system, is used to assess the severity of vascular invasion. It is a five-point grading system derived by comparing the morphologic features in MRI with histopathological references.

Score 0: Pattern of tumor extension through the rectal wall is not nodular; no adjacent vessels. It uses basic criteria for scoring that are as follows:

Score 1: Minimal extramural stranding; no adjacent vessels.

Score 2: Stranding in proximity of vessels but no tumor signal in normal caliber lumen.

Score 3: Intermediate signal in lumen of vessels; slight vessel expansion.

Score 4: Irregular vessel contour; definite tumor signal.

**2. MRI and CT scan parameters**

All MRI examinations were performed with a 3.0-T MRI scanner (Discovery 750W®, GE Healthcare, Waukesha, WI), and CT scans were obtained using a 256-detector row MDCT scanner (Revolution Xtream®, GE Healthcare, Waukesha, WI). The specific scanning parameters are shown in Table S1 and Table S2.

**Table S1. MRI scan parameter list**

| Sequence name | TR(ms) | TE(ms) | slice thickness(mm) | slice space(mm) | FOV(mm) | acquisition matrix | number of excitations |
| --- | --- | --- | --- | --- | --- | --- | --- |
| T1WI | 4694 | 102 | 5 | 1 | 380 | 320 × 224 | 2 |
| T2WI(axial) | 4435 | 102 | 5 | 1 | 380 | 320 × 224 | 2 |
| T2WI(oblique axial) | 4500 | 102 | 3 | 0.5 | 256 | 200 × 220 | 4 |
| T2WI(sagittal) | 4500 | 102 | 3 | 0.5 | 256 | 200 × 220 | 4 |
| DWI | 4243 | 73.4 | 4 | 1 | 320 | 128 × 130 | 2 |
| CET1WI | 4.6 | 1.1 | 2 | 0 | 380 | 260 × 224 | 1 |

**Table S2. CT scan parameter list**

| Scan mode | Tube voltage(kVp) | Tube current (mA) | Gantry rotation speed (s/r) | Pitch | Width of detector (mm) | FOV(mm) | Slice thickness (mm) | Slice gap (mm) |
| --- | --- | --- | --- | --- | --- | --- | --- | --- |
| Plain CT scan | 120 | 150-550 | 0.5 | 0.992 | 160 | 360 | 5 | 5 |
| Contrast enhancement CT scan | 120 | 150-550 | 0.5 | 0.992 | 160 | 360 | 5 | 5 |

**Ⅱ. Information of Dimension reduction**

**1. Standardization of data**

Extracted texture features were standardized, which removed the unit limits of the data of each feature and converted it into a dimensionless pure value. This allowed the indexes of different units or orders to be compared and weighted. We used a z-score normalization to make the image intensities fit a standard normal distribution with $\mu\text{=0}$ and $\sigma\text{=1}$, where $\mu$ is the mean value of the images, and $\sigma$ is the standard deviation. The normalized values (also called z-scores) of the image intensities (*x*) were calculated as follows:

$$z=\frac{x-\mu}{\sigma}$$

After image z-score normalization, the number of radiomics features arrived at 378 according to AK software. Radiomics features included the histogram (42 features), Haralick (10 features), Formfactor (9 features), Gray-Level Co-occurrence Matrix (126 features, GLCM), Run length matrix (180 features, RLM) and Gray Level Size Zone Matrix(11 features, GLSZM). The feature details are described in the table below.


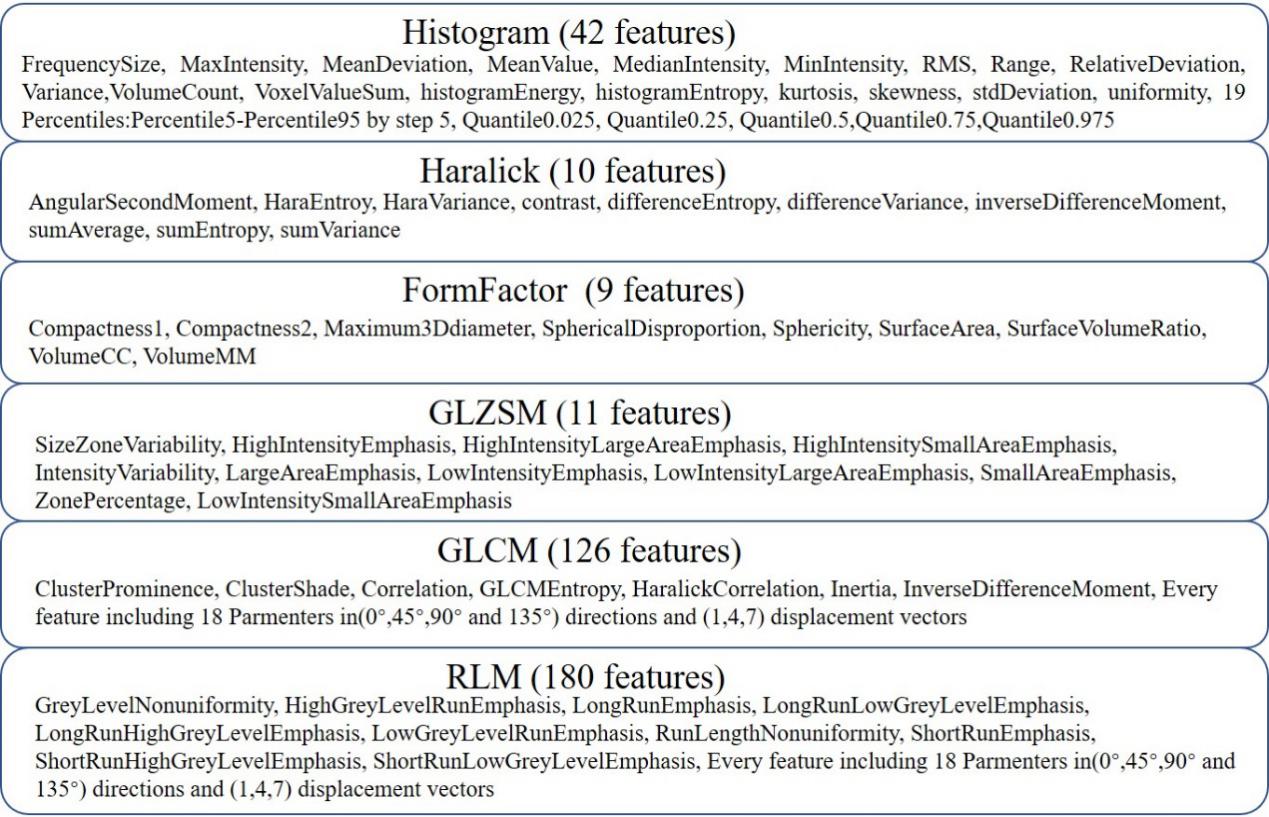


1. **Details on Dimension reduction**

378 radiomics features were extracted from each patient's enhanced CT, T2WI and CE-T1WI, respectively. 287, 302 and 298 features were retained by detection of robustness and reproducibility, respectively. The mRMR algorithm was used to select the features that had the greatest correlation with the outcome of EMVI, and these features with the least redundancy were then selected in surplus features and an optimal subset of complementary predictive features was built. In this study, the optimal feature sets composed of 42, 36 and 48 features are obtained by mRMR algorithm， respectively. Second, dimensionality reduction of the selected optimal feature sets was performed using the least absolute shrinkage and selection operator (LASSO) method. LASSO is a powerful algorithm for regression analysis with high dimensional predictors. The LASSO algorithm shrinks some coefficients and reduces others to exactly 0 via an absolute constraint. Thus, LASSO is an outstanding method for feature selection as it retains good features using both a subset selection and ridge regression. In this study, LASSO selected 16, 20 and 19 nonzero coefficients from enhanced CT, T2WI and CE-T1WI, respectively. Finally, we make a redundancy analysis of these selected features, and the results show that in each mode image, the correlation between all features does not exceed 0.7. Figure S1 shows the dimension reduction process of LASSO. Figure S2 show heatmap of correlation coefficients. Details of these features are shown in Table S3.


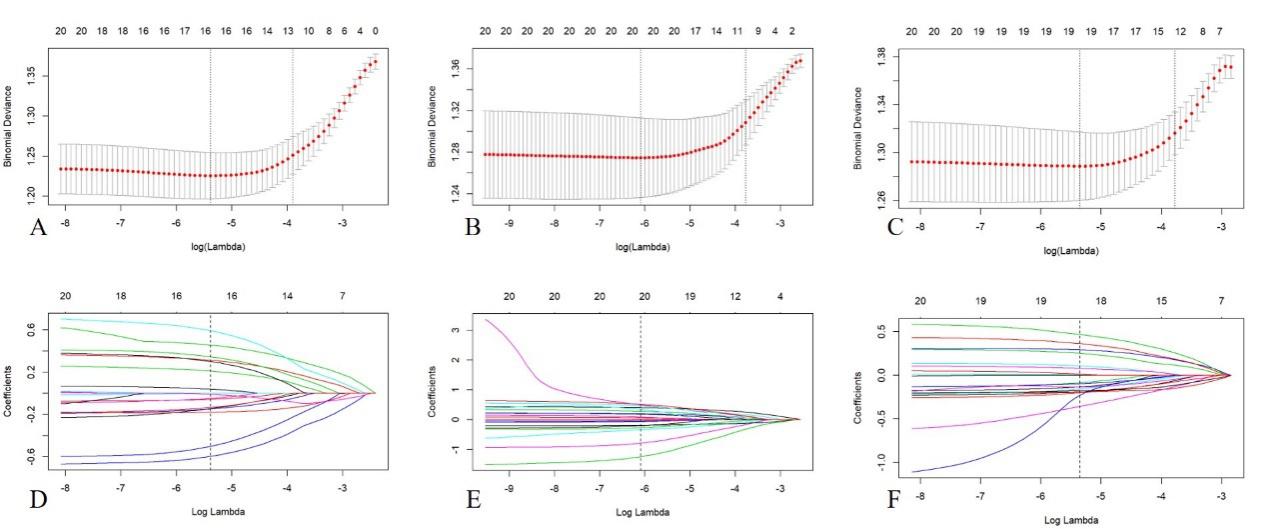


Figure S1. Feature selection using LASSO logistic regression of enhanced CT(A,D), T2WI(B,E) and CE-T1WI(C,F). A 10-fold cross-validation was applied with the regularization parameter ($l$) of the LASSO regression model and selected when the deviance was minimal (A, B, C). Coefficients are plotted against the log (l) sequence. Ultimately, 16, 20 and 19 nonzero coefficients were selected (B, E, F). LASSO, least absolute shrinkage and selection operation.


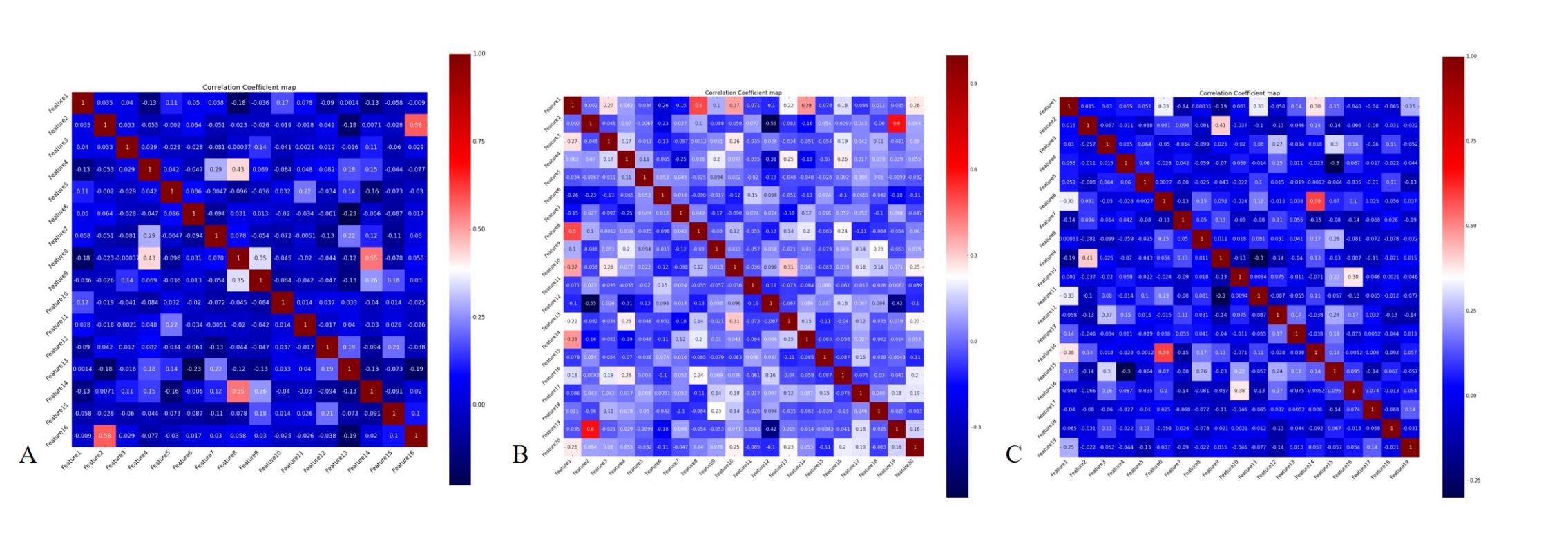


Figure S2. Heatmap show the correlation coefficients between the remaining features after dimensionality reduction from enhanced CT (A), T2WI (B) and CE-T1WI (C), respectively.

**Table S3. The classification and calculation formula of radiomics features**

| **Category** | **Feature** | **VIF** | **Intraclass correlation coefficient （95%CI）** | **Formula** | **Describe** |
| --- | --- | --- | --- | --- | --- |
| GLCM | GLCMEntropy_angle135_0)ffset7 | 1.494 | 0.834(0.812-0.882) | $-\sum_{ij} g(i,j){log}_{2}(i,j)$ | Entropy is a measure of randomness of intensity image. Entropy shows the amount of information of the image that is needed for the image compression. Entropy measures the loss of information or message in a transmitted signal and also measures the image information. |
|  | GLCMEntropy_angle45_offset1 | 1.07 | 0.865(0.846-0.891) |  |  |
|  | GLCMEntropy_AllDirection_offset1 | 3.124 | 0.812(0.769-0.843) |  |  |
|  | GLCMEntropy_AllDirection_offset1_SD | 4.131 | 0.885(0.842-0.903) |  |  |
|  | GLCMEntropy_AllDirection_offset4_SD | 1.323 | 0.941(0.902-0.976) |  |  |
|  | GLCMEntropy_AllDirection_offset7 | 2.273 | 0.83(0.811-0.859) |  |  |
|  | GLCMEntropy_AllDirection_offset7_SD | 3.166 | 0.823(0.79-0.863) |  |  |
|  | Inertia_AllDirection_offset1_SD | 1.459 | 0.875(0.833-0.911) | $\sum_{i,j} ((i-j)^{2}g(i,j))$ | It reflects the clarity of the image and texture groove depth. The contrast is proportional to the texture groove, high values of the groove produces more clarity, in contrast small values of the groove will result in small contrast and fuzzy image. |
|  | InverseDifferenceMoment_AllDirection_offset4_SD | 1.365 | 0.886(0.849-0.914) | $\sum\sum\frac{1}{1+{(i-j)}^{2}}g(i,j)$ | Inverse Difference Moment (IDM) is the local homogeneity. It is high when local gray level is uniform and inverse GLCM is high. IDM weight value is the inverse of the Contrast weight. |
|  | Correlation_angle135_offset7 | 2.087 | 0.823(0.776-0.843) | $-\sum_{i,j} \frac{(i-\mu)(j-\mu)g(i,j)}{\sigma^{2}}$ | Image-based Correlation measures the similarity of the grey levels in neighboring pixels, tells how correlated a pixel is to its neighbor over the whole image.  Range = [-1 1]. Correlation is 1 or -1 for a perfectly positively or negatively correlated image |
|  | Correlation_AllDirection_offset7_SD | 2.295 | 0.896(0.872-0.947) |  |  |
|  | ClusterShade_AllDirection_offset4_SD | 1.265 | 0.831(0.811-0.879) | $\sum_{i,j} ((i-\mu)+(j-\mu))^{3}g(i,j)$ | Cluster analysis or clustering is the task of grouping a set of objects in such a way that objects in the same group (cluster) are more similar (in some sense or another) to each other than to those in other groups (clusters). It is a common technique for statistical data analysis. |
|  | ClusterShade_AllDirection_offset7_SD | 2.52 | 0.856(0.829-0.893) |  |  |
|  | ClusterShade_AllDirection_offset1_SD | 2.618 | 0.834(0.796-0.866) |  |  |
|  | ClusterShade_angle90_offset7 | 1.577 | 0.868(0.848-0.931) |  |  |
| RLM | LongRunLowGreyLevelEmphasis_AllDirection_offset1_SD | 1.887 | 0.878(0.843-0.924) | $\frac{1}{n_{r}}\sum_{j=i}^{N} \sum_{i=1}^{M} \frac{p(i,j,\theta)j^{2}}{i^{2}}$ | The grey level run-length matrix (RLM) 𝐏𝐫(𝐢, 𝐣 | 𝛉 ) is defined as the numbers of runs with pixels of gray level i and run length j for a given direction θ. RLMs is generated for each sample image segment having directions (0°,45°,90° &135°) |
|  | LongRunLowGreyLevelEmphasis_AllDirection_offset7_SD | 2.395 | 0.913(0.871-0.926) |  |  |
|  | LongRunLowGreyLevelEmphasis_AllDirection_offset4_SD | 1.918 | 0.906(0.884-0.941) |  |  |
|  | LongRunLowGreyLevelEmphasis_angle135_offset7 | 3.854 | 0.828(0.804-0.847) |  |  |
|  | LongRunLowGreyLevelEmphasis_angle90_offset7 | 1.131 | 0.848(0.811-0.884) |  |  |
|  | ShortRunHighGreyLevelEmphasis_angle90_offset4 | 1.253 | 0.845(0.821-0.903) | $\frac{1}{n_{r}}\sum_{j=i}^{N} \sum_{i=1}^{M} \frac{p(i,j,\theta)i^{2}}{j^{2}}$ |  |
|  | ShortRunHighGreyLevelEmphasis_AllDirection_offset7_SD | 1.822 | 0.805(0.763-0.824) |  |  |
|  | RunLengthNonuniformity_AllDirection_offset7_SD | 1.746 | 0.869(0.844-0.943) | $\frac{1}{n_{r}}\sum_{j=i}^{N} \sum_{i=1}^{M} p(i,j,\theta))^{2}$ |  |
|  | RunLengthNonuniformity_AllDirection_offset1_SD | 1.996 | 0.849(0.828-0.887) |  |  |
|  | GreyLevelNonuniformity_AllDirection_offset7_SD | 2.102 | 0.884(0.878-0.933) |  |  |
|  | HighGreyLevelRunEmphasis_AllDirection_offset7_SD | 1.821 | 0.834(0.802-0.863) | $\frac{1}{n_{r}}\sum_{j=i}^{N} \sum_{i=1}^{M} p(i,j,\theta)i^{2}$ |  |
|  | HighGreyLevelRunEmphasis_AllDirection_offset4_SD | 1.452 | 0.818(0.773-0.846) |  |  |
|  | LowGreyLevelEmphasis_AllDirection_offset1_SD | 1.368 | 0.883(0.852-0.953) | $\frac{1}{n_{r}}\sum_{j=i}^{N} \sum_{i=1}^{M} \frac{p(i,j,\theta)}{i^{2}}$ |  |
|  | LowGreyLevelEmphasis_AllDirection_offset4_SD | 2.892 | 0.877(0.851-0.911) |  |  |
|  | LowGreyLevelRunEmphasis_AllDirection_offset1_SD | 1.632 | 0.873(0.844-0.942) |  |  |
|  | LongRunEmphasis_AllDirection_offset1_SD | 1.039 | 0.893(0.863-0.957) | $\frac{1}{n_{r}}\sum_{j=i}^{N} \sum_{i=1}^{M} p(i,j,\theta)j^{2}$ |  |
|  | LongRunEmphasis_AllDirection_offset7_SD | 2.294 | 0.833(0.793-0.896) |  |  |
|  | LongRunHighGreyLevelEmphasis_AllDirection_offset4_SD | 1.389 | 0.838(0.815-0.862) | $\frac{1}{n_{r}}\sum_{j=i}^{N} \sum_{i=1}^{M} p(i,j,\theta)i^{2}j^{2}$ |  |
|  | ShortRunLowGreyLevelEmphasis_AllDirection_offset4_SD | 1.97 | 0.855(0.832-0.865) | $\frac{1}{n_{r}}\sum_{j=i}^{N} \sum_{i=1}^{M} \frac{p(i,j,\theta)}{i^{2}j^{2}}$ |  |
| Haralick | HaralickCorrelation_ALLDirection_0ffset4_SD | 1.105 | 0.883(0.851-0.933) | -  where $u_{t}$ and$\sigma_{t}$ are the mean and standard deviation of the row (or column, due to symmetry) sums. | Measures the degree of similarity of the gray level of the image in the row or column direction. Represents the local grey level correlation, the greater its value, the greater the correlation |
| FormFactor | SufaceVolumeRatio | 2.788 | 0.861(0.809-0.923) | $\sum_{i=1}^{N} \frac{1}{2}\left\vert a_{i}b_{i}\times a_{i}b_{i} \right\vert$ | These group of features includes descriptors of the three-dimensional size and shape of the tumor region. Let in the following definitions 𝑉 denote the volume and 𝐴 the surface area of the volume of interest. |
|  | Compactness2 | 1.871 | 0.815(0.783-0.866) | $36\pi\frac{V^{2}}{A^{3}}$ |  |
|  | VolumeMM | 2.34 | 0.932(0.869-0.964) | The maximum 3D diameter, surface area and volume provide information on the size of the lesion. Measures of compactness, spherical disproportion, sphericity and the surface to volume ratio describe how spherical, rounded, or elongated the shape of the tumor is. |  |

VIF: variance inflation factor
